# Influence of dietary crude protein content on fattening performance and nitrogen excretion of Holstein steers

**DOI:** 10.1111/asj.13438

**Published:** 2020-08-05

**Authors:** Mitsuru Kamiya, Tomoya Yamada, Mikito Higuchi

**Affiliations:** ^1^ Division of Livestock Feeding and Management Central Region Agricultural Research Center NARO Tochigi Nasushiobara Japan

**Keywords:** crude protein, fattening, Holstein, nitrogen excretion, steers

## Abstract

The objective was to investigate the influence of crude protein (CP) content in a fattening diet on feed intake, body weight gain, nitrogen excretion, and carcass traits in Holstein steers. Steers (initial body weight 241 ± 26 kg) consumed feed with the following CP content: (a) 17.7% during the early period (from 7 to 10 months of age) and 13.9% during the late period (from 11 to 18 months of age) (HIGH, *n* = 3), and (b) 16.2% during the early period and 12.2% during the late period (LOW, *n* = 4). The CP intake was lower in the LOW than the HIGH group. Urinary and total nitrogen excretion in the late period tended to be lower (*p* < .10) in the LOW than the HIGH group. However, growth performance and carcass traits were not affected by dietary CP content. Free histidine and total amino acid contents in the *longissimus thoracis* muscle tended to be higher (*p* < .10) in the HIGH than the LOW group, however, the CP contents were not affected by dietary CP content. The results of this experiment suggest that decreasing dietary CP to 16% (early period) or 12% (late period) of dry matter would reduce nitrogen excretion from Holstein fattening farms without affecting productivity.

## 1 INTRODUCTION

Reducing the environmental impact of the livestock industry has become an important issue. In particular, the reduction in greenhouse gas (GHG) emissions is required worldwide. Nitrous oxide is a GHG that is generated from livestock manure (Petersen, 2018; Powers & Capelari, 2016). The emission of nitrous oxide is reduced by decreasing the amount of urinary nitrogen (N) excretion associated with dietary crude protein (CP) level (Bao, Zhou, & Zhao, 2018). Therefore, decreasing the dietary CP content to the appropriate level is important for minimizing environmental impacts such as GHG and ammonia emissions without reducing productivity.

Several studies have reported on methods of reducing N excretion (Aemiro et al., 2019; Lee, Morris, Lefever, & Dieter, 2019; Zhou et al., 2019). For fattening beef cattle, decreasing the dietary CP reduces the amount of N excreted in crossbred steers (Vasconcelos et al., 2009) and Black Angus heifers (Koenig & Beauchemin, 2013). In contrast, Holstein steers, the major fattening cattle in Japan, have a higher body growth rate and higher CP requirements than other breeds (National Agriculture and Food Research Organization, 2009). In Japan, the CP content of the diet for fattening cattle is generally higher than the required amount, and Holstein steers are fed a fattening diet with relatively high CP content (National Agriculture and Food Research Organization, 2009). From the relationships of N intake and urinary N excretion of fattening Holstein steers based on reports (Choumei, Terada, & Hirooka, 2006; Terada, Abe, Nishida, & Shibata, 1998), it is possible that decreasing the dietary CP content can reduce N excretion, especially urinary N excretion on Japanese cattle fattening farms. However, no studies have examined the effects of decreasing dietary CP content on fattening performance and N excretion of Holstein steers in recent fattening programs in Japan.

The object of this study was to investigate the influence of CP content in a fattening diet on the feed intake, body weight gain, carcass traits, and N excretion of Holstein steers.

## 2 MATERIALS AND METHODS

### 2.1 Cattle and feeding management

Seven Holstein steers (average age: 7 months) were assigned to a high‐CP (HIGH, average body weight (BW) 230 kg, *n* = 3) or a low‐CP (LOW, average BW 249 kg, *n* = 4) concentrate feeding group. Steers were kept on average from 7 to 18 months of age, including an early period (from 7 to 10 months of age) and a late period (from 11 to 18 months of age). Steers were typically group fed in pens at 7 and 8 months of age and fed individually from 9 to 18 months of age. BW was measured weekly before morning feeding. At an average of 18 months of age, steers were slaughtered in a commercial slaughter facility. Carcass traits were evaluated according to standard procedures of the Japan Meat Grading Association. All animal treatments were approved by the Animal Care and Use Committee of the Institute of Livestock and Grassland Science, NARO.

The chemical composition and ingredients of the diet are presented in Table 1. Steers were fed the concentrate mix and hay according to the feeding program shown in Table 2. The concentrate mix was offered in the morning (around 9o' clock) and evening (around 16o' clock). Hay was offered in the morning. Water and mineral blocks were available ad libitum. Refusals were collected and measured daily before morning feeding.

**TABLE 1:** Chemical composition and ingredients of the diet

	Concentrate mix for early period		Concentrate mix for late period		Timothy hay	Orchardgrass hay
	HIGH	LOW	HIGH	LOW		
Dry matter, % of FM	88.5	88.3	88.6	88.5	88.0	85.7
Chemical composition, % of DM						
TDN	79.8	80.2	81.9	82.3	54.4	50.1
Crude protein	20.0	18.1	14.5	12.5	8.6	7.4
Acid detergent fiber	10.2	10.4	8.1	7.3	34.5	37.0
Neutral detergent fiber	23.2	23.8	23.9	23.8	60.2	64.7
Ingredients, % of FM						
Corn	33.2	39.2	18.8	24.6	–	–
Barley	–	–	44.0	44.0	–	–
Rice	3.0	3.0	1.0	1.0	–	–
Wheat bran	15.0	15.0	20.0	20.0	–	–
Corn gluten feed	11.7	11.7	3.6	3.6	–	–
Soybean meal	13.0	7.0	6.0	0.9	–	–
Rapeseed meal	–	–	1.2	0.5	–	–
Alfalfa hay cube	7.0	7.0	–	–	–	–
Alfalfa meal	3.0	3.0	–	–	–	–
Other by‐products^a^	9.7	9.7	4.0	4.0	–	–
Supplement^b^	4.4	4.4	1.4	1.4	–	–

Abbreviations: DM, dry matter; FM, fresh matter; HIGH, dietary CP contents of consumed feed were 17.7% during the early period and 13.9% during the late period; LOW, dietary CP contents of consumed feed were 16.2% during the early period and 12.2% during the late period; TDN, TDN estimated according to Standard Tables of Feed Composition in Japan (NARO, 2010); TDN, total digestible nutrients.

^a^ Rice bran, defatted rice bran, barley bran with hull and polish, DDGS, corn gluten meal.

^b^ Cane molasses, minerals, vitamines, and feed additives.

**TABLE 2:** Feeding program for fattening Holstein steers

	Months of age											
	7	8	9	10	11	12	13	14	15	16	17	18
Concentrate mix for early period, kgFM/day	6.0–6.8	7.0–7.8	8.0–8.5	9.0–9.5	10.0^a^							
Concentrate mix for late period, kg/FM/day						10.0–10.3	10.5	10.8	11.0	11.3	11.5	11.8
Timothy hay, kgFM/day	2.0	2.0	2.0	2.0	2.0^b^							
Orchardgrass hay, kgFM/day						1.6	1.6	1.6	1.6	1.6	1.6	1.6

Note

Values are the upper limit.

Abbreviation: FM, fresh matter.

^a^ Mixture of the concentrate mix for early period and the concentrate mix for the late period.

^b^ Mmixture of timothy hay and orchardgrass hay.

The dry matter contents of feed were determined by air‐forced drying at 105°C for 16 hr. The feed samples were analyzed for N content using the macro‐Kjeldahl method and the fiber content was determined using the detergent method. The total digestible nutrients (TDN) of the diet were estimated based on the values in the Standard Tables of Feed Composition in Japan (National Agriculture and Food Research Organization, 2010).

### 2.2 Blood and muscle samples

Blood samples for measuring urea N were taken from the jugular vein into a heparinized tube every month before morning feeding from 6 months of age before the experiment to 18 months of age. Plasma was separated by centrifugation at 1,760 *g* for 20 min at 4°C and stored at −30°C until analyzed. The plasma urea N concentration was determined by the urease‐GLDH method (Sanritsu Co., Ltd, Chiba, Japan). The *longissimus thoracis* (LT) muscle around the 7th rib was taken from the carcass and reserved in a refrigerator at 4°C until 14 days after slaughter. The muscle samples were analyzed for moisture, crude fat, and CP contents. Free amino acids (AA) in muscles were determined using an automatic AA analyzer (JLC‐500/V; JEOL Ltd., Tokyo, Japan) after deproteinization by the addition of 5% perchloric acid.

### 2.3 Nitrogen balance trials

After acclimatizing the steers to the facilities and feeds in advance, steers moved to individual pens or stanchion stall, and then the N balance trials were conducted. To measure the N excretion and retention at 9 months of age in the early period, steers were kept for 7 days in individual pens, including the last 3 days sampling period. Steers were given up to 8.5 kg of a concentrate mix and up to 2 kg of timothy hay daily. Water was available ad libitum. At 13 months of age in the late period, steers were kept for 11 days in a stanchion stall, including the last 3 days sampling period. Steers were given up to 10.5 kg of a concentrate mix and up to 1.6 kg of orchardgrass hay daily. Water was available ad libitum.

Spot urine and feces samples were collected from all steers in the morning and evening during the sampling period. Urine and feces samples were analyzed for N content using the macro‐Kjeldahl method. Urinary creatinine concentrations were determined by the Jaffe method (LabAssay Creatinine; Wako Pure Chemical Industries, Ltd, Osaka, Japan). Urine volume was estimated using the creatinine concentration as an internal marker. The daily urinary excretion of creatinine was estimated to be 22.8 mg/kg of the BW (Asai et al., 2005). Therefore, urinary N excretion was estimated as follows.

UrinaryNexcretion(g/day)=22.8×BW÷Cr×UN

where BW is the body weight, kg; Cr is the urinary creatinine concentration, mg/L; and UN is the urinary N concentration, g/L.

The feed and fecal acid‐insoluble ash (AIA) concentration was analyzed by the 4 N HCl method (TeradaIwasaki, Tano, & Haryu, 1979). The fecal weight was estimated using the AIA concentration as an internal marker. Therefore, the fecal N excretion was estimated as follows.

FecalNexcretion(g/day)=AI÷(FA÷100)×(FN÷100)

where AI is the AIA intake, g; FA is the fecal AIA concentration, %; and FN is the fecal N concentration, %.

### 2.4 Statistical analysis

Differences in all data were examined by ANOVA with the dietary treatment as a factor, supported by SAS Add‐In 7.1 for Microsoft Office (SAS Institute Japan Ltd., Tokyo, Japan). Values of *p* < .05 and *p* < .10 were considered to indicate significance and tendency, respectively.

## 3 RESULTS

There were significant differences (*p* < .01) in CP intake among treatments, and the dietary CP content of consumed feed was 17.7% for the HIGH group or 16.2% for the LOW group in the early period, and 13.9% for the HIGH group or 12.2% for the LOW group in the late period (Table 3). In the early period, the dry matter intake (DMI) and TDN were significantly higher (*p* < .01) by 0.1 kg/day in the LOW group, but the dietary CP content did not affect the DMI and TDN in the late and total period (Table 3). There were no feed differences in BW, average daily gain, and gain‐to‐feed ratio (Table 3).

**TABLE 3:** Influence of dietary crude protein content on body weight and feed intake of Holstein steers

	HIGH	LOW	*p*‐value
Body weight, kg			
Initial (early period)	230 ± 30	249 ± 24	.40
Initial (late period)	401 ± 25	421 ± 29	.39
Final	719 ± 14	717 ± 55	.94
Average daily gain, kg/day			
Early period	1.45 ± 0.08	1.46 ± 0.15	.91
Late period	1.37 ± 0.17	1.27 ± 0.15	.45
Total	1.40 ± 0.12	1.34 ± 0.14	.58
Dry matter intake, kg/day			
Early period	8.4 ± 0.0	8.5 ± 0.0	<.01
Late period	10.5 ± 0.3	10.6 ± 0.3	.92
Total	9.8 ± 0.2	9.9 ± 0.2	.71
Gain to feed ratio			
Early period	0.172 ± 0.009	0.171 ± 0.017	.90
Late period	0.130 ± 0.012	0.120 ± 0.011	.34
Total	0.142 ± 0.010	0.135 ± 0.012	.46
TDN intake, kg/ day			
Early period	6.3 ± 0.0	6.4 ± 0.0	<.01
Late period	8.2 ± 0.3	8.3 ± 0.2	.79
Total	7.6 ± 0.2	7.6 ± 0.2	.58
CP intake, kg/day			
Early period	1.49 ± 0.00	1.38 ± 0.00	<.01
Late period	1.47 ± 0.05	1.29 ± 0.04	<.01
Total	1.48 ± 0.03	1.32 ± 0.03	<.01
CP % of consumed feed			
Early period	17.7 ± 0.1	16.2 ± 0.0	<.01
Late period	13.9 ± 0.1	12.2 ± 0.0	<.01

Note

CP % of consumed feed: This value represents the percentage of CP intake relative to dry matter intake during the early or late period.

Values are expressed as mean ± *SD*.

**FIGURE 1:**
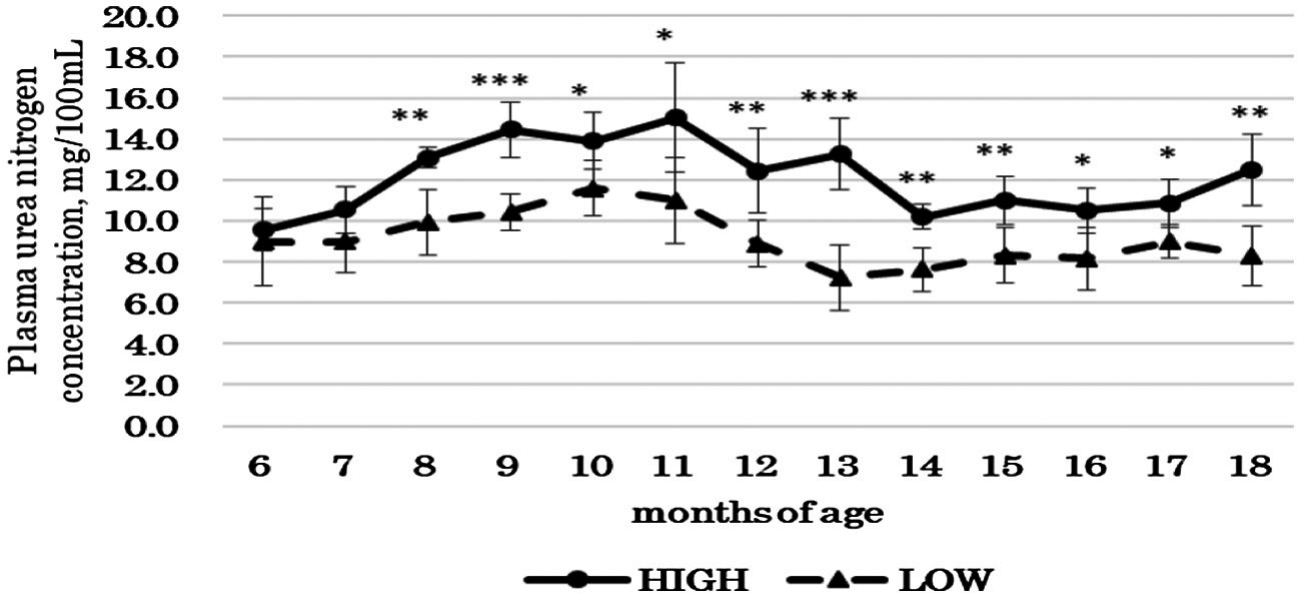
Influence of dietary crude protein content on plasma urea nitrogen concentration of Holstein steers. HIGH (●, *n* = 3): Dietary CP content of consumed feed was 17.7% during the early period (7–10 months of age) and 13.9% during the late period (11–18 months of age). LOW (▲, *n* = 4): Dietary CP contents of consumed feed were 16.2% during the early period and 12.2% during the late period. Values are expressed as mean ± *SD*. * *p* < .10, ** *p* < .05, *** *p* < .01 between the HIGH and LOW group

The plasma urea N concentration tended to be low or significantly lower in the LOW group from 8 to 18 months of age (Figure 1). Regarding the N balance trial, there was a difference only in the N intake in the early period (Table 4), but in the late period, decreasing the dietary CP content significantly reduced the N intake (*p* < .01) and tended to reduce (*p* < .10) urinary N excretion and total N excretion (Table 5).

**TABLE 4:** Influence of dietary crude protein content on fecal and urinary nitrogen of Holstein steers in the early period (9 months of age)

	HIGH	LOW	*p*‐value
Nitrogen balance trial, g/day			
Nitrogen intake	241.0 ± 1.5	234.8 ± 2.3	<.01
Fecal nitrogen excretion	82.6 ± 5.1	77.5 ± 12.5	.54
Urinary nitrogen excretion	106.7 ± 8.6	99.3 ± 8.4	.31
Total nitrogen excretion	189.3 ± 5.3	176.9 ± 16.1	.27
Nitrogen retension	51.8 ± 4.5	58.0 ± 14.8	.52
Nitrogen balance trial, g/MBW			
Nitrogen intake	3.00 ± 0.14	2.79 ± 0.14	.11
Fecal nitrogen excretion	1.03 ± 0.05	0.93 ± 0.19	.41
Urinary nitrogen excretion	1.33 ± 0.14	1.18 ± 0.09	.13
Total nitrogen excretion	2.36 ± 0.11	2.10 ± 0.25	.17
Nitrogen retension	0.65 ± 0.07	0.69 ± 0.17	.71

Note

Values are expressed as mean ± *SD*.

Abbreviations: HIGH: dietary CP content of consumed feed was 17.7% during the early period; Low: dietary CP content of consumed feed was 16.2% during the early period; MBW, metabolic body weight.

**TABLE 5:** Influence of dietary crude protein content on fecal and urinary nitrogen of Holstein steers in the late period (13 months of age)

	HIGH	LOW	*p*‐value
Nitrogen balance trial, g/day			
Nitrogen intake	226.0 ± 10.5	188.9 ± 12.5	<.01
Fecal nitrogen excretion	65.6 ± 10.0	70.4 ± 7.8	.51
Urinary nitrogen excretion	111.3 ± 15.4	85.1 ± 16.8	<.10
Total nitrogen excretion	176.9 ± 5.7	155.5 ± 15.1	<.10
Nitrogen retension	49.1 ± 15.7	33.4 ± 11.7	.19
Nitrogen balance trial, g/MBW			
Nitrogen intake	2.05 ± 0.07	1.68 ± 0.11	<.01
Fecal nitrogen excretion	0.59 ± 0.08	0.63 ± 0.09	.60
Urinary nitrogen excretion	1.01 ± 0.16	0.76 ± 0.12	<.10
Total nitrogen excretion	1.60 ± 0.09	1.38 ± 0.10	<.05
Nitrogen retension	0.44 ± 0.14	0.30 ± 0.11	.18

Note

Values are expressed as mean ± *SD*.

Abbreviations: high: dietary CP content of consumed feed was 13.9% during the late period; low: dietary CP content of consumed feed was 12.2% during the late period; MBW, metabolic body weight.

Data related to productivity, such as carcass weight, rib‐eye area, rib thickness, subcutaneous fat thickness, and yield score, were not affected by dietary CP content (Table 6). The dietary CP content did not change the meat quality, such as the beef marbling standard, beef color standard, beef fat color standard, beef meat brightness, beef meat firmness (SHIMARI), beef meat texture (KIME), beef fat brightness, and quality (Table 6).

**TABLE 6:** Influence of dietary crude protein content on carcass and meat traits of Holstein steers

	HIGH	LOW	*p*‐value
Carcass			
Carcass weight, kg	381 ± 10	391 ± 28	.59
Rib eye area, cm^2^	39.3 ± 4.0	36.3 ± 4.8	.41
Rib thickness, cm	4.9 ± 0.2	4.9 ± 0.3	.97
Subcutaneous fat thickness, cm	1.7 ± 0.4	1.8 ± 0.6	.76
Yield score	69.5 ± 0.8	68.9 ± 1.0	.41
BMS no.	2.0 ± 0.0	2.0 ± 0.0	–
BCS no.	4.0 ± 0.0	4.0 ± 0.0	–
BFS no.	3.0 ± 0.0	3.0 ± 0.0	–
Beef meat brightness	2.0 ± 0.0	2.5 ± 0.6	.20
Beef meat firmness (SHIMARI)	2.0 ± 0.0	2.0 ± 0.0	–
Beef meat texture (KIME)	3.0 ± 0.0	3.0 ± 0.0	–
Beef fat brightness and quality	4.0 ± 0.0	3.8 ± 0.5	0.44
Longissimus thoracis muscle at the 6−7th cross section			
Moisture, %	66.4 ± 1.1	64.6 ± 1.2	0.10
Crude fat, %	13.0 ± 1.7	14.9 ± 1.5	0.17
Crude protein, %	20.1 ± 1.1	19.9 ± 0.3	0.77
Free amino acids, mg/100 g			
Total amino acids	279.0 ± 25.0	242.3 ± 19.7	<0.10
Total essential amino acids	66.1 ± 4.1	58.8 ± 7.3	0.19
Arginine	12.3 ± 2.2	10.0 ± 1.1	0.12
Histidine	4.5 ± 0.6	3.5 ± 0.5	<0.10
Isoleucine	6.1 ± 0.1	5.8 ± 0.9	0.61
Leucine	10.1 ± 0.3	9.8 ± 1.4	0.72
Lysine	9.2 ± 1.8	7.5 ± 0.8	0.16
Methionine	5.0 ± 0.3	4.6 ± 0.8	0.39
Phenylalanine	6.0 ± 0.4	5.6 ± 0.8	0.43
Throleonine	5.7 ± 0.8	5.1 ± 0.7	0.32
Valine	7.0 ± 0.2	6.7 ± 0.7	0.48
Total non‐essential amino acids	213.0 ± 20.9	183.5 ± 16.5	<0.10
Alanine	33.5 ± 4.7	32.3 ± 3.4	0.71
Asparagine	10.3 ± 1.5	8.8 ± 0.9	0.15
Aspartic acid	0.4 ± 0.1	0.5 ± 0.1	0.23
Citrulline	2.7 ± 0.8	3.0 ± 0.4	0.66
Glutamic acid	6.0 ± 1.2	5.4 ± 0.8	0.51
Glutamine	129.2 ± 21.8	106.0 ± 13.5	0.14
Glycine	11.2 ± 1.1	9.9 ± 0.6	0.10
Ornithine	1.3 ± 0.6	0.9 ± 0.2	0.38
Proline	3.3 ± 0.5	2.8 ± 0.3	0.13
Serine	8.7 ± 1.4	7.8 ± 1.3	0.41
Tyrosine	6.3 ± 0.4	6.0 ± 1.0	0.62

Note

Values are expressed as mean ± *SD*.

Abbreviations: BCS, beef color standard; BFS, beef fat color standard; BMS, beef marbling standard; HIGH, dietary CP contents of consumed feed were 17.7% during the early period and 13.9% during the late period; LOW, dietary CP contents of consumed feed were 16.2% during the early period and 12.2% during the late period.

The moisture, crude fat, and CP contents of the LT muscle around the 7th cross section were not affected by dietary CP content (Table 6). Although there were no statistical differences in many free AA contents of the LT muscle, the free histidine, total AA, and total non‐essential AA contents tended to be higher (*p* < .10) in the HIGH group than in the LOW group (Table 6).

## 4 DISCUSSION

In this experiment, the average gain was about 1.4 kg/day during the total period and the weight gain was as fast as for typical fattening Holstein steers. Steers in both treatments were fed almost the same amount with the same roughage. Two types of concentrate mix with almost the same TDN content and different CP contents were used in the experiment with different ratios of corn, soybean meal, and rapeseed meal. The concentrate mix intake was similar to that of typical fattening Holstein steers. Therefore, according to the recent fattening program in Japan, the effects of the dietary CP content on the fattening performance of Holstein steers with fast weight gain and high CP requirements were investigated in this experiment.

Growing beef cattle have higher weight gain when the dietary CP content is high (Braman, Hatfield, Owens, & Lewis, 1973; Byers & Moxon, 1980; Haskins, Wise, Craig, & Barrick, 1967; Perry, Shields, Dunn, & Mohler, 1983). In this experiment, the early period was a conversion period from the growing phase to the fattening phase, but there was no effect of dietary CP content on weight gain. On the other hand, the results of dietary CP content on fattening performance were different among past experiments researching the fattening of beef cattle. In research regarding the fattening of beef cattle from 300 kg to 500 or 600 kg, Cole, Defoor, Galyean, Duff, and Gleghorn (2006) reported that dietary CP content of 11.5% produces the same productivity as 13%, and Gleghorn et al. (2004) recommended a dietary CP content of 13%, as compared to 11.5% and 14.5%. In this experiment, the dietary CP content was 12.2% in the LOW group and 13.9% in the HIGH group when the BW was between 400 and 700 kg during the late period, and the productivity was not affected.

Nitrogen excretion is expected to decrease when the dietary CP content is lowered. In fattening heifers weighing 500 kg or more, the amount of urea N excretion decreased with a CP content of 13% as compared to 14.5% (Koenig & Beauchemin, 2013). Similarly, in this experiment, urinary N and total N excretion decreased by lowering the dietary CP content in the late period. The difference in total N excretion was mainly related to the difference in urinary N excretion, since there was no effect on fecal N excretion. Therefore, it is considered that total N excretion was higher in the HIGH group than in the LOW group due to increased urinary N excretion caused by excess rumen ammonia or excess AA in the body associated with excessive N intake. On the other hand, the difference in N intake during the N balance trial in the early period was smaller than the difference in the CP intake throughout the early period because the dietary CP content of the HIGH group used in the N balance trial was lower than usual. As a result, there was no difference in N excretion during the early period in this experiment. However, with the same results as in other reports (Cole et al., 2005; Vasconcelos et al., 2009), plasma urea N concentration was lowered by decreasing the dietary CP content. It is well known that the plasma urea N concentration is highly related to N excretion (Kohn, Dinneen, & Russek‐Cohen, 2005), and it was estimated that N excretion during the early period would also decrease in the LOW group. Therefore, it is expected that N excretion can be reduced by decreasing the dietary CP content to an appropriate value throughout the fattening period.

Considering the results of the N balance trial and the CP intake during the feeding period, the total N excretion during the fattening period is about 10% lower in the LOW group than in the HIGH group. In the GHG inventory of Japan, nitrous oxide emissions are calculated by multiplying the amount of N contained in manure by the emission factor for each type of manure treatment method (Ministry of the Environment, Japan, and Greenhouse Gas Inventory Office of Japan (GIO), Center for Global Environmental Research (CGER), National Institute for Environmental Studies (NIES), 2020). If the manure treatment method is the same, it is expected that nitrous oxide emissions will be reduced by about 10% in the LOW group as compared to the HIGH group, as well as total N excretion.

The free AA content in meat is focused on as taste components of meat and influenced by storage conditions (Watanabe, Ueda, & Higuchi, 2004). Moreover, Koutsidis et al. (2008) reported that the difference of free AA content in meat between a beef breed and a dairy breed was small, but the effect of feed was significant. Iwamoto, Iwaki, and Oka (2010) reported that the dietary CP content in the early fattening period did not affect the free AA content in the LT muscle. On the other hand, the effect of differences in dietary CP content throughout the fattening period on beef free AA content has not been reported in the past. In the present study, most free AA contents of the LT muscle were not affected by the dietary CP content, but free histidine, total AA, and total non‐essential AA tended to be lower in the LOW group than in the HIGH group. Ueda et al. (2007) reported that most free AA concentrations in meat were negatively correlated with fat content. In this study, the average crude fat content in meat was 13% in the HIGH group and 14.9% in the LOW group. The protein content in meat was 20.1% in the HIGH group and 19.9% in the LOW group. Ueda et al. (2007) reported a negative correlation between fat content and free AA content even in low‐fat meat (<23%) with a relatively constant protein content. Therefore, in the present study, dietary CP content affected the free total AA content in meat, but fat content in meat may also have affected. The free AA are related to the taste of meat (Watanabe et al., 2004), and histidine has a bitter taste (Kawai, Sekine‐Hayakawa, Okiyama, & Ninomiya, 2012). However, most AA contents, including histidine, are not expected to exceed the taste threshold (Schiffman, Sennewald, & Gagnon, 1981). Therefore, it is not clear whether the difference in AA of this experiment had a significant effect on the actual taste. In terms of productivity, carcass traits, such as carcass weight and meat quality, were similar with both treatments. Therefore, it was considered that the dietary CP content for fattening Holstein steers does not need to be higher than 16% in the early period and 12% in the late period.

The GHG reduction is important for the sustainability of the beef industry (Gleason & White, 2019), and GHG is reduced by decreasing excessive N excretion (Eckard, Grainger, & Klein, 2010). Generally, the dietary CP content is set higher than the CP requirement for fattening cattle in Japan. In this experiment, dietary CP contents of 16% for the early period and 12% for the late period were sufficient to reduce N excretion without affecting productivity. These results suggest that the dietary CP content of fattening Holstein steers should be reduced to at least this experimental level. The results of this experiment also suggest that N excretion can be reduced from cattle fattening farms in Japan, which contributes to GHG reduction.
